# Supplementary material for: DMRforPairs: identifying Differentially Methylated Regions between unique samples using array based methylation profiles
Source: BMC Bioinformatics. 2014 May 15;15:141. doi: 10.1186/1471-2105-15-141 (PMC4046028; doi:10.1186/1471-2105-15-141)
Supplement: Additional file 2 — DMRforPairs output for the comparison of A431-MCF7 and NA17018-NA17105. Please start from the HTML files in each folder. Available via the BMC Bioinformatics website. [file 1471-2105-15-141-S2.zip › 1394847754114233_MOESM2_ESM/A431_MCF7/figures/6562.pdf]

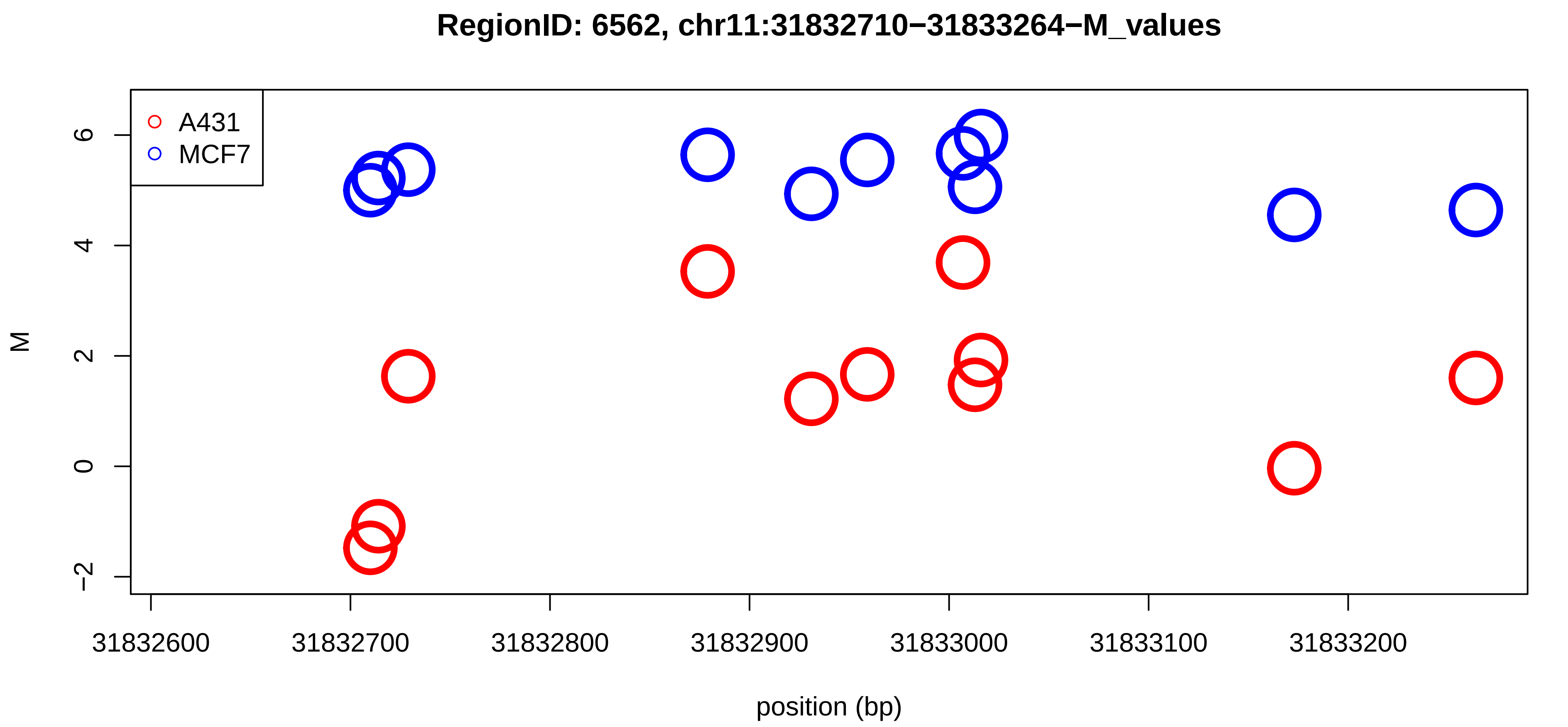

RegionID: 6562, chr11:31832710−31833264−M_values
A431
MCF7
M
−2
0
2
4
6
31832600
31832700
31832800
31832900
31833000
31833100
31833200
position (bp)

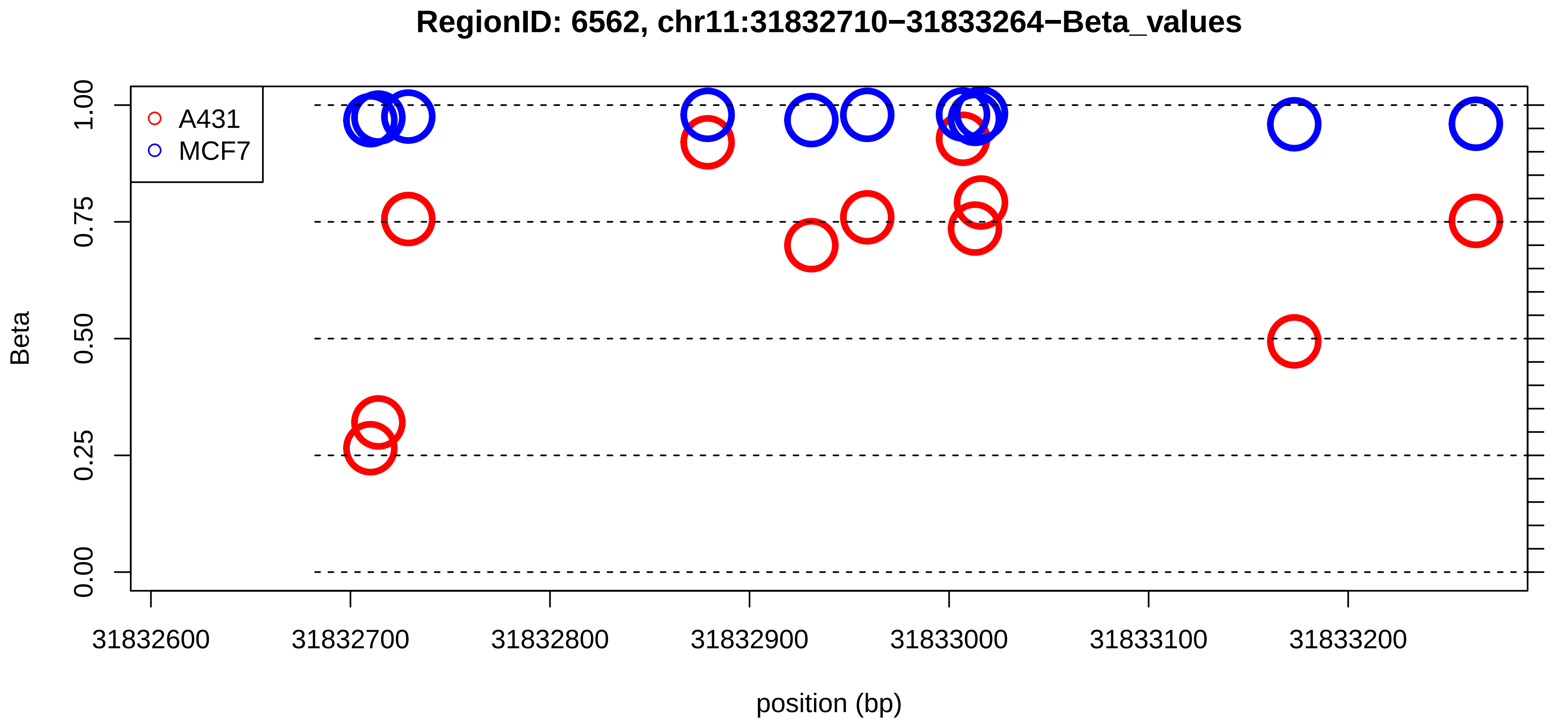

RegionID: 6562, chr11:31832710−31833264−Beta_values
A431
MCF7
Beta
0.00
0.25
0.50
0.75
1.00
31832600
31832700
31832800
31832900
31833000
31833100
31833200
position (bp)

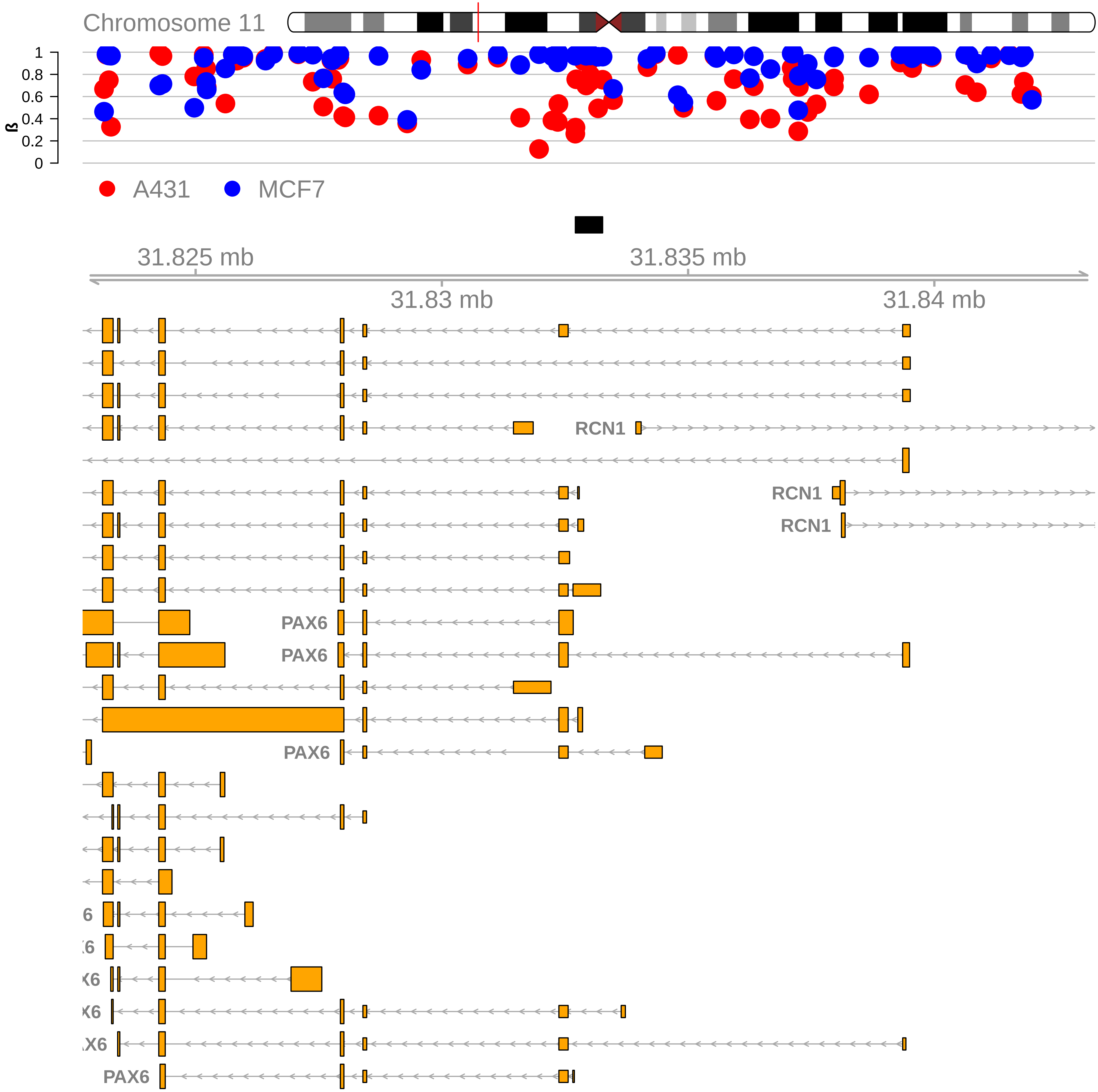

Chromosome 11
ß
1
0.8
0.6
0.4
0.2
0
A431
MCF7
31.825 mb
31.835 mb
31.83 mb
31.84 mb
RCN1
RCN1
RCN1
PAX6
PAX6
PAX6
PAX6
